# Factors influencing coping skills of middle-aged adults in COVID-19, South Korea

**DOI:** 10.3389/fpubh.2024.1248472

**Published:** 2024-02-01

**Authors:** Minkyung Gu, Heeyoung Woo, Sohyune Sok

**Affiliations:** ^1^Department of Nursing, College of Health Science, Daejin University, Pocheon-si, Gyeonggi-do, Republic of Korea; ^2^Department of Nursing, Sahmyook Health University, Seoul, Republic of Korea; ^3^College of Nursing Science, Kyung Hee University, Seoul, Republic of Korea

**Keywords:** COVID-19, coping, health, family support, impact of event

## Abstract

**Background:**

In light of the persistent COVID-19 pandemic, there is a compelling imperative to enhance the COVID-19 coping capacity among middle-aged adults within the South Korean population. Consequently, there is a need for further research endeavors in this area.

**Objective:**

This study aims to explore and identify the factors influencing the coping skills of middle-aged adults in COVID-19, South Korea.

**Methods:**

This study used a cross-sectional descriptive design. Participants were 147 middle-aged adults living in Seoul, Geounggi-do, and Chungchung-do in South Korea. Data included demographics, coping skills, the impact of the event, perceived health status, psychological wellbeing, and family support. The data were analyzed using the SPSS Statistics 25.0 program. Data were collected from March to July 2020.

**Results:**

The regression model was significant (*F* = 13.56, *p* < 0.001), and the adjusted *R*-squared representing the explanatory power of the model was 0.63. The strongest predictor was perceived health status (β = 0.34), followed by family support (β = 0.31), impact of event (β = −0.24), underlying disease (β = −0.13), and economic status (β = 0.11).

**Conclusion:**

This study suggests that to improve the coping skills of middle-aged adults with COVID-19, their perceived health status and family support should be strengthened, and the impact of the event should be decreased. Also, it needs that underlying diseases are managed and economic status is improved. Nurses need to pay more attention to the influencing factors to improve the coping skills of middle-aged adults in COVID-19, South Korea.

## Introduction

Middle-aged adulthood is the pre-stage of old age, transitioning from the first half of life to the second half of life. In middle-aged adulthood, the change in consciousness coexists with the role performance and the behavioral style in which life changes occur. Moreover, it is a time when one establishes his own values and finds emotional stability by achieving a sense of social accomplishment (1, 2). On the other hand, middle-aged adults often have unresolved problems in relation to their physical and self-perceived health status according to their developmental stage, stress on their children, ability to care for older adult parents, and relationships with their families (3, 4). In the transition period in one's life, middle-aged men may experience negative thoughts that may lead to maladaptive behaviors and isolation from society due to professional loss and retirement (1, 5). Furthermore, middle-aged women experience psychological changes related to menopause, such as nervousness, depression, and anxiety (6, 7). After all, since middle-aged adults have to take on these various roles, they are prone to chronic diseases and are decisively in a period of a health crisis (3, 4, 6–8). Therefore, the ripple effect on the meaning of life is much greater than in any age group.

Meanwhile, COVID-19, which first appeared in December 2019, is known as a respiratory infectious disease that is characterized by asymptomatic spread and high infectivity. It is known that an individual infected with COVID-19 spreads the virus to 2.2 individuals, and the fatality rate was also found to have reached the level of 4% or more (9). COVID-19 poses a real threat to the survival of middle-aged adults and causes disarray in their lives (10). Additionally, as middle-aged adults face the uncertain COVID-19 event, they are unable to control themselves, and their emotions become very intense in this regard (11). Finally, the COVID-19 infection reduces the driving force for life in middle-aged adults and acts as a negative factor in developing coping skills (12–14).

In general, developing coping skills among middle-aged adults is a key task mediated by psychological adaptation as they experience a crisis, i.e., the COVID-19 infection (1). In other words, for middle-aged adults, coping skills include all cognitive and behavioral traits that seek to address the internal and external needs of life (12, 13). Therefore, how they accept life changes amid COVID-19 can contribute to forming more stable coping skills (1). The spread of COVID-19 can be an event that further encourages them to consider their self-perceived health status with an unstable feeling of youth loss (15). It may lead to psychological decline, depression, and anxiety and, ultimately, adversely affect the psychological wellbeing of middle-aged adults (3, 5, 6, 8, 16). Moreover, the COVID-19 pandemic is already negatively impacting the economic status of our society, leading to a continuous decrease in household income, disrupting the family support system for middle-aged adults, and aggravating the anxiety of middle-aged adults (15, 17). As the COVID-19 pandemic continues, there is an urgent need to strengthen the capacity of middle-aged Koreans to cope with COVID-19. Therefore, consistent research efforts in this area are needed, and in this regard, this study is significant.

The purpose of this study was to examine and identify the factors influencing the coping skills of middle-aged adults in COVID-19, South Korea. The aims were (1) to identify the four additional factors related to coping skills among middle-aged adults in the context of COVID-19 in South Korea and (2) to examine the predictive effect or the association of the general characteristics of study participants with coping skills.

As a hypothesis, the significant factors influencing coping skills would be the impact of the event, perceived health status, psychological wellbeing, and family support.

## Methods

### Study population

A cross-sectional descriptive design was used. Participants were a total of 147 middle-aged adults living in Seoul, Geounggi-do, and Chungchung-do in South Korea. Eligibility criteria were middle-aged adults between 40 and 60 years of age who understood the purpose of the study by having clear consciousness and being capable of verbal and non-verbal communication. Subjects excluded from this study were those who had previously been medically diagnosed with a psychiatric disorder or were currently taking psychotropic medications. They were excluded for the sake of the reliability of this study. The number of subjects was calculated with a significance level of 0.05 and a median effect size of 0.15 in order to secure 95.0% of the statistical power for regression analysis of the G^*^Power 3.1.5 program (18). Based on this, the appropriate sample size was 138. Considering the dropout rate of 10.0%, 152 middle-aged adults were asked to fill out a questionnaire. The number of samples in this study was finally used for data analysis of 147 middle-aged adults (96.7%), excluding five middle-aged adults who had non-response and missing questionnaires.

Participants in this study were 83 men (56.5%) and 64 women (43.5%). As for age, 44 subjects (33.3%) were between 55 and 59 years old. The rate of living with children was the highest at 74 subjects (50.3%), followed by the rate of living alone with a spouse at 55 subjects (37.4%). As for underlying diseases, 85 subjects (57.8%) did not have underlying diseases, which was higher than 62 subjects (42.2%) who had such underlying diseases. Finally, in this study, 123 subjects (83.7%) were well-observing of the COVID-19 quarantine rules, accounting for the majority (Table 1).

**Table 1:** General characteristics of study participants.

**Characteristics**	***n***	**%**
**Gender**		
Male	83	56.5
Female	64	43.5
**Age (years)**		
< 45	35	20.6
45–49	37	25.1
50–54	31	21.0
55–59	44	33.3
**Education**		
Middle and high school	41	27.9
University	52	35.4
Graduate school ≤	54	36.7
**Marital status**		
Married	122	83.0
Divorced/widowed	13	8.8
Single	12	8.2
**Economic status**		
Good	21	14.3
Moderate	105	71.4
Bad	21	14.3
**Living together**		
Son or daughter	74	50.3
Husband or wife	55	37.4
Living alone	12	8.2
Others	6	4.1
**Monthly income (10,000 Won)**		
< 200	33	22.4
200–400	53	36.1
400≥	61	41.5
**Smoking**		
No	11	7.5
Less than one pack of cigarettes	37	25.2
One pack or more cigarettes	99	87.3
**Drinking**		
No	59	40.1
2 times a month≥	21	14.3
3–4 times a month ≤	33	22.4
2 times a week≥	28	19.0
3–4 times a week ≤	6	4.1
**Leisure activity**		
Yes	68	46.3
No	79	53.7
**Underlying disease**		
Yes	62	42.2
No	85	57.8
**COVID-19 quarantine rules**		
Yes	123	83.7
No	24	16.3

### Measurements

#### General characteristics of study participants

General characteristics of study participants based on a literature review and previous research (3–8, 15, 16) included gender, age, education, marital status, economic status, living together, monthly income, smoking, drinking, leisure activity, underlying disease, and COVID-19 quarantine rules. This consisted of a total of 12 items. The economic status was the self-perceived economic level, which was divided into good, moderate, and bad, and was answered by self-report. Living together refers to people currently in cohabitation. Leisure activities refer to all activities done by an individual of his or her own free will, such as taking a break from one's duties, participating in the community, volunteering, or developing one's abilities. The underlying diseases were chronic diseases such as cardiovascular disease, chronic respiratory disease, and diabetes.

#### Coping skills

For coping skills, Shin and Kim's instrument (19), which was made according to the Korean situation by revising and supplementing the Coping Strategy Indicator (CSI) developed by Amirkan (20), was used. This instrument is composed of three subdimensions, such as seeking social support, problem-solving, and avoidance, and consists of a total of 33 items. As a 5-point Likert scale, the score ranges from a minimum of 33 to a maximum of 165, and a higher score means a higher level of coping skills in the subject. The Coping Strategy Indicator (CSI) showed acceptable content validity in the previous study (19). At the time of the revision (19), Cronbach's α = 0.84, and reliability in this study was Cronbach's α = 0.93.

#### Impact of the event

In terms of the impact of an event, the Impact of Event Scale (IES) developed by Horowitz et al. (21), and translated by Eun et al. (22), was used. This instrument consists of a total of 22 items, including emotions or feelings at the time that remind you of the event, actions that remind you of the event, and socially restricted activities or physical responses to the event. As a 5-point Likert scale, the score ranges from a minimum of 22 to a maximum of 110, and a higher score means a higher impact of the event. Concerning the Impact of Event Scale (IES), at the time of the revision (22), Cronbach's α = 0.89, and reliability in this study was Cronbach's α = 0.94.

#### Perceived health status

Perceived health status refers to the subjective health status as perceived by the person himself/herself, and the instrument used was developed by Ware (23), Speake et al. (24), and translated by Cho (25). This instrument consists of a total of three items regarding one's current health status, degree of interference with daily life, and comparison of one's health status with the same age group. As a 5-point Likert scale, the score ranges from a minimum of 3 to a maximum of 15, and a higher score means a higher perceived health status of the subject. As for the perceived health status scale, at the time of the revision (25), Cronbach's α = 0.92, and reliability in this study was Cronbach's α = 0.89.

#### Psychological wellbeing

In terms of psychological wellbeing, Park's instrument (26), which was translated to fit the Korean situation, was used. It consists of a total of 45 items with six dimensions (self-acceptance, positive relations with others, autonomy, environmental mastery, purpose in life, and personal growth) used by Ryff (27). The items of the instrument included “I am influenced by other people's opinions,” “I do not have a warm and reliable relationship with other people,” “It is difficult to maintain close relationships with other people,” and “I think I have done everything that I had to do in my life.” As a 5-point Likert scale, the score ranges from a minimum of 45 to a maximum of 225, and a higher score means higher psychological wellbeing. The psychological wellbeing scale, at the time of the revision (26), had Cronbach's α = 0.91, and reliability in this study was Cronbach's α = 0.93.

#### Family support

In terms of family support, Cobb's (28) instrument used by Kang and Han (29) was used in this study. It consists of a total of 11 items, including “My family gives me courage and encouragement to recover.” “I can trust and rely on my family,” and “My family is dedicated to helping me.” As a 5-point Likert scale, the score ranges from a minimum of 11 to a maximum of 55, and a higher score means higher family support. Regarding the family support scale, at the time of the revision (29), Cronbach's α = 0.87, and reliability in this study was Cronbach's α = 0.95.

### Data collection

In this study, data were collected from March to July 2020. Study participants were selected using convenience sampling from a break room with small groups of visitors and employees of a church, a general hospital, and public institution workers located in Seoul, Geounggi-do, and Chungchung-do in South Korea. They were invited to a quiet office located in a church, hospital, or institution for data collection. The researcher explained the purpose of the study directly to the study participants and had middle-aged adults fill out the questionnaire to collect data after giving consent to voluntary participation. For the safety and reliability of data collection, the questionnaire was distributed to middle-aged adults while properly wearing a mask, observing social distancing, and washing their hands. It took about 25 min or more for one study participant to fill out the questionnaire.

### Ethical considerations

After this study had been approved by the Institutional Review Board of D University (IRB No. 1040656-201912-SB-02-12, approval date: 7 February 2020) the study participants were informed that it would not be used for any purpose other than the research purpose by asking the study participants for voluntary cooperation in data collection. In addition, it was explained that the subject's anonymity and confidentiality were communicated to them, explaining that they could withdraw at any time during the study if they did not want to.

### Data analysis

Data were analyzed using the SPSS Statistics 25.0 (IBM Inc., Armonk, NY, United States) program. Descriptive statistics (frequency, percentage, mean, and standard deviation) were used to analyze the general characteristics of the study participants and the levels of study variables. An independent *t*-test and an ANOVA (*F*-test) were used to examine the differences in coping skills according to the general characteristics of the study participants. The Scheffe test was used for the *post-hoc* test. Pearson's correlation coefficient was used to analyze the correlations among study variables related to coping skills. Among the general characteristics of the study participants, statistics on nominal and continuous variables were obtained through linear regression analysis. Hierarchal stepwise multiple regression analysis was used to examine factors influencing coping skills. The normality of the data distribution was analyzed by the Shapiro-Wilk test. A *p*-value of < 0.05 was considered statistically significant.

## Results

### Levels of coping skills, impact of the event, perceived health status, psychological wellbeing, and family support

The levels of coping skills of study participants were 51.42 points, and the impact of the event was 72.94 points. Perceived health status was 10.22 points, psychological wellbeing was 151.41 points, and family support was 41.03 points (Table 2). The levels of variables were slightly higher than the median value.

**Table 2:** Levels of coping skills, impact of event, perceived health status, psychological wellbeing, and family support.

**Variables**	**Range point (median value)**	**Min**.	**Max**.	**Total score average (Mean ±SD)**
Coping skills	33–165 (51.00)	33.00	80.00	51.42 ± 11.56
Impact of event	22–110 (72.00)	39.00	107.00	72.94 ± 13.44
Perceived health status	3–15 (10.00)	4.00	15.00	10.22 ± 2.36
Psychological wellbeing	45–225 (150.00)	94.00	219.00	151.41 ± 18.96
Family support	11–55 (41.00)	13.00	55.00	41.03 ± 9.89

### Differences in coping skills according to the general characteristics of study participants

The levels of coping skills according to the general characteristics of the study participants showed statistically significant differences in economic status (*F* = 18.17, *p* < 0.001), monthly income (*F* = 12.92, *p* < 0.001), leisure activity (*t* = −2.96, *p* = 0.004), the underlying disease (*t* = −2.99, *p* = 0.003), and the COVID-19 quarantine rules (*t* = 2.56, *p* = 0.012).

Through the study, it can be seen that the higher the economic status and monthly average income of the study participants, the better their skills to cope with COVID-19. In particular, it can be seen that the coping skills of the study participants who do not engage in leisure activity, do not have underlying diseases, and are well observing the COVID-19 quarantine rules were good (Table 3).

**Table 3:** Differences on coping skills according to the general characteristics of study participants.

**Characteristics**	**Mean ±SD**	**Independent *t*-test or *F*-test (*p*) Scheffe *post-hoc* test**
**Gender**		
Male	52.59 ± 12.61	1.41 (0.161)
Female	49.89 ± 9.92	
**Age (years)**		
< 45	50.55 ± 10.92	1.25 (0.181)
45–49	51.63 ± 16.17	
50–54	49.58 ± 9.23	
55–59	59.78 ± 11.78	
**Education**		
Middle and high school	50.61 ± 10.11	1.00 (0.370)
University	23.23 ± 11.65	
Graduate school ≤	50.28 ± 12.46	
**Marital status**		
Married	50.96 ± 11.10	1.02 (0.364)
Divorced/widowed	55.77 ± 12.15	
Single	51.33 ± 15.24	
**Economic status**		
Good	62.90 ± 12.06	18.17 (< 0.001^*^) a > b,c
Moderate	50.55 ± 9.90	
Bad	44.24 ± 11.14	
**Living together**		
Son or daughter	51.07 ± 10.51	0.50 (0.686)
Husband or wife	51.27 ± 11.73	
Living alone	55.17 ± 16.74	
Others	49.50 ± 12.23	
**Monthly income (10,000 Won)**		
< 200	48.54 ± 10.57	12.92 (< 0.001^*^) c > a,b
200–400	49.55 ± 9.27	
400≥	59.73 ± 12.96	
**Smoking**		
No	54.78 ± 12.70	2.71 (0.070)
Less than one pack of cigarettes	46.82 ± 10.24	
One pack or more cigarettes	50.67 ± 11.04	
**Drinking**		
No	51.20 ± 9.18	1.06 (0.379)
2 times a month≥	52.38 ± 14.12	
3–4 times a month ≤	49.03 ± 11.40	
2 times a week≥	52.36 ± 12.49	
3–4 times a week ≤	58.83 ± 18.50	
**Leisure activity**		
Yes	48.46 ± 10.76	−2.96 (0.004^*^)
No	53.96 ± 11.69	
**Underlying disease**		
Yes	48.16 ± 11.06	−2.99 (0.003^*^)
No	53.79 ± 11.40	
**COVID-19 quarantine rules**		
Yes	75.03 ± 10.68	2.56 (0.012^*^)
No	45.54 ± 13.16	

^*^p < 0.05.

### Correlations between coping skills and factors related to it

The correlation between coping skills and the impact of an event (*r* = −0.63) showed a significant negative correlation, and correlations between perceived health status (*r* = 0.64), psychological wellbeing (*r* = 0.49), and family support (*r* = 0.63) showed significant positive correlations.

Taken together, it can be seen that the better the participant's skills to cope with COVID-19, the lower the impact of the COVID-19 event. In addition, it can be seen that the better the participant's skills to cope with COVID-19, the higher the perceived health status, psychological wellbeing, and family support of the subject (Table 4).

**Table 4:** Correlations between coping skills and factors related it.

**Variables**	**Coping skills**	**Impact of event**	**Perceived health status**	**Psychological wellbeing**	**Family support**
	**Pearson correlation coefficient** ***r***				
Coping skills	1				
Impact of event	−0.63^**^	1			
Perceived health status	0.64^**^	−0.62^**^	1		
Psychological wellbeing	0.49^**^	−0.50^**^	0.49^**^	1	
Family support	0.63^**^	−0.56^**^	0.42^**^	0.47^**^	1

^**^p < 0.01.

### Factors influencing coping skills

As a result of testing the assumptions of the regression analysis, it was found that all the assumptions of the regression equation were satisfied. Tolerance of multicollinearity was 0.37–0.88, which was over 0.10, and the Variance Inflation Factor (VIF) was 1.19–2.67, which was not >10, so all variables did not have a problem in terms of multicollinearity. Also, the correlations between the independent variables influencing the subject's coping skills were 0.42–0.64, which were < 0.80, so all variables were independent of each other.

The factors influencing the coping skills of the subjects were analyzed by using hierarchical multiple regression. As a result, the first-step regression model with general characteristics was statistically significant (*F* = 3.66, *p* < 0.001). The variable that was statistically significant in the first step was economic status (β = 0.32, *p* = 0.002), and the explanatory power of the first step regression model was 25.0%. In the second step, the main variables, the impact of the event, and perceived health status were added. The variables that were statistically significant in the second step (*F* = 12.09, *p* < 0.001) with the subject's general characteristics and two main variables were economic status (β = 0.20, *p* = 0.001), underlying disease (β = −0.15, *p* = 0.054), the impact of the event (β = −0.42, *p* < 0.001), and perceived health status (β = 0.38, *p* < 0.001). The explanatory power increased by 31.0% compared to the first step. In the third step (*F* = 13.56, *p* < 0.001), psychological wellbeing and family support were added to complete the regression model. Variables that were statistically significant in the third step were economic status (β = 0.11, *p* = 0.016), underlying disease (β = −0.13, *p* = 0.048), impact of event (β = −0.24, *p* = 0.002), perceived health status (β = 0.34, *p* < 0.001), and family support (β = 0.31, *p* < 0.001). Furthermore, the explanatory power increased by 7.0% in the third step, compared to the second step. Taken together, among the factors influencing the coping skills of middle-aged adults amid the COVID-19 pandemic, the most significant variable was perceived health status, followed by family support, the impact of the event, the underlying disease, and economic status. The explanatory power of the final regression model was 63.0% (Table 5).

**Table 5:** Factors influencing coping skills.

**Variables**	***B***	**SE**	**β**	***t***	***p***
**Stage 1**					
Gender	0.03	0.16	0.01	0.17	0.869
Age (years)	−1.97	2.32	−0.09	−0.85	0.396
Education	0.20	1.20	0.01	0.16	0.870
Marital status	−1.26	1.16	−0.09	−1.09	0.279
Economic status	6.95	2.15	0.32	3.24	0.002
Living together	−0.07	0.92	−0.01	−0.08	0.940
Monthly income (10,000 Won)	−1.93	1.49	−0.13	−1.30	0.197
Smoking	−0.14	1.31	−0.01	−0.11	0.913
Drinking	−0.99	0.90	−0.11	−1.09	0.276
Leisure activity	2.33	2.06	0.10	1.13	0.259
Underlying disease	1.19	2.22	0.05	0.53	0.595
COVID-19 quarantine rules	3.08	2.37	0.12	1.30	0.196
Adj *R*^2^ = 0.25, *F* = 3.66, *p* < 0.001					
**Stage 2**					
Gender	0.13	0.13	0.07	1.02	0.311
Age (years)	0.24	1.81	0.01	0.13	0.894
Education	1.28	0.93	0.09	1.37	0.172
Marital status	−0.54	0.90	−0.04	−0.60	0.549
Economic status	4.36	1.67	0.20	2.61	0.001
Living together	0.19	0.71	0.02	0.26	0.793
Monthly income (10,000 Won)	−0.31	1.16	−0.02	−0.27	0.788
Smoking	−0.09	1.01	−0.01	−0.09	0.931
Drinking	−0.82	0.69	−0.09	−1.18	0.240
Leisure activity	−1.05	1.62	−0.05	−0.65	0.518
Underlying disease	−3.51	1.82	−0.15	−1.93	0.054
COVID-19 quarantine rules	1.33	1.84	0.05	0.72	0.471
Impact of event	−0.36	0.07	−0.42	−4.92	< 0.001
Perceived health status	−1.88	0.37	0.38	−5.08	< 0.001
Adj *R*^2^ = 0.56, *F* = 12.09, *p* < 0.001					
**Stage 3**					
Gender	0.06	0.12	0.03	0.53	0.597
Age (years)	−0.58	1.69	−0.03	−0.34	0.734
Education	1.23	0.88	0.09	1.41	0.162
Marital status	−0.18	0.84	−0.01	−0.22	0.830
Economic status	2.27	1.62	0.11	1.40	0.016^*^
Living together	−0.19	0.67	−0.02	−0.28	0.783
Monthly income (10,000 Won)	−0.30	1.08	−0.02	−0.28	0.784
Smoking	−0.01	0.94	−0.01	−0.01	0.994
Drinking	−0.63	0.65	−0.07	−0.97	0.336
Leisure activity	0.03	1.53	0.01	0.02	0.987
Underlying disease	−2.92	1.70	−0.13	−1.72	0.048^*^
COVID-19 quarantine rules	1.20	1.72	0.05	0.70	0.486
Impact of event	−0.20	0.08	−0.24	−2.70	0.002^*^
Perceived health status	−1.66	0.36	0.34	−4.65	< 0.001^*^
Psychological wellbeing	−0.04	0.04	−0.07	−1.05	0.294
Family support	−0.36	0.09	0.31	−4.21	< 0.001^*^
Adj *R*^2^ = 0.63, *F* = 13.56, *p* < 0.001					

^*^p < 0.05.

## Discussion

When faced with a stressful situation, active coping skills lead to desirable adaptation as a positive source, which can be used as a support resource to help individuals with maladaptation and strengthen endurance when facing a difficult situation. The level of coping skills of middle-aged adults who are participating in this study was close to the median value, and it is similar to the results of Chung's (8) and Chae and Joung's (30) study. Therefore, it is necessary to develop and utilize an intervention program that improves the coping skills of middle-aged adults amid the COVID-19 pandemic and helps them to respond flexibly according to individual circumstances.

Next, in this study, it can be seen that middle-aged adults with higher economic status and monthly average income have better coping skills. This result is similar to the previous studies (8, 30), in which a decrease in economic power among middle-aged adults can be a factor that induces stress, lowers self-esteem, and acts as a negative factor in their lives. Therefore, for middle-aged adults to develop effective coping skills when facing difficult situations, it is necessary to help them positively overcome crises by establishing and institutionalizing active measures to support and utilize the physical resources of local communities under the leadership of the government. Furthermore, in this study, it was found that middle-aged adults without an underlying disease who were adhering to the COVID-19 quarantine rules had better coping skills. The result supports the research findings that one's own health beliefs are more important in preserving one's health than those of a subject with an underlying disease amid the COVID-19 pandemic (31, 32). In other words, the results are similar to those of previous studies in which coping skills and health status vary according to the degree of sensitivity and severity as judged by an individual in a crisis such as the COVID-19 infection (13, 14, 16, 31–33). Therefore, it is necessary to periodically utilize mental health programs to help form desirable health beliefs for middle-aged adults amid the COVID-19 pandemic.

In this study, in terms of the correlations between the coping skills of middle-aged adults and major variables, a subject's coping skills were stronger when the impact of the event was low and the perceived health status, psychological wellbeing, and family support were high. This was similar to the study results of Shin and Baek (32), who reported that middle-aged adults who perceived their current health status as average or worse felt a high sense of crisis amid the COVID-19 pandemic. Through this study, it is considered that the sharing of correct information should be expanded to help middle-aged adults discern wrong information on their risk perception of COVID-19 infection and to actively engage middle-aged adults in public health programs by establishing public health partnerships and public relations with the central and local governments (34, 35).

Furthermore, the factors influencing the coping skills of middle-aged adults appeared in the order of perceived health status, family support, the impact of the event, the underlying disease, and economic status. Middle-aged adulthood is an age group that should be actively engaged in economic activities since they can be responsible for the livelihood of the family. Moreover, in modern society, the family structure is changing and is disconnected due to rapid industrialization (1, 3, 6, 8). At this time when the cohesion of family members is gradually weakening, this study shows that positive thinking, in which they perceive themselves as being healthy, and family support are most important for the coping skills of middle-aged adults, as they rely on personal inner strength. In other words, in a crisis such as the COVID-19 pandemic, continuous care of family members is more important than anything else for middle-aged adults to develop coping skills. This can be attributed to the fact that the overall emotion of middle-aged adults can be expressed to encompass all social and economic factors, as well as physical and psychological factors. Therefore, middle-aged adults should manage their perceived health better and should be able to maintain psychological wellbeing to actively cope with the COVID-19 crisis. Likewise, it is necessary to strengthen ties not only with immediate family members but also with relatives, friends, co-workers, and the community to overcome the COVID-19 crisis through positive thinking (36).

### Implications for practice, policy, and research

Based on this study, a strategy to improve the coping skills of middle-aged adults and overcome the COVID-19 pandemic is to increase the perceived health status of middle-aged adults and to maintain their psychological wellbeing to positively overcome the crisis. In particular, it is necessary to strengthen their relationships with family members so that they can overcome the COVID-19 crisis. Nurses should actively consider these influencing factors in nursing interventions for middle-aged adults amid the COVID-19 pandemic. Moreover, developing and applying a coping skills improvement program for middle-aged adults, which includes these influencing factors, will make it possible to prevent COVID-19 infection and maintain the health of middle-aged adults. In the future, a qualitative study is needed to fundamentally understand and analyze the inner world of middle-aged adults regarding the COVID-19 pandemic. Furthermore, experimental research is needed to expand the factors influencing the coping skills of middle-aged adults, develop a nursing intervention program that addresses the coping skills of Korean middle-aged adults amid the COVID-19 pandemic, and verify its effectiveness.

### Limitations

The subjects were extracted through convenience sampling of middle-aged adults living in certain areas of Korea. In other words, data collection was varied based on convenience sampling and not having a dedicated quiet space. There were also gender disparities as the majority of them were males. Therefore, there are limitations in expanding the study results to explain the factors influencing the coping skills of all middle-aged adults during the COVID-19 pandemic. The fact that various social and cultural factors were not considered may be a research limitation. Additionally, cross-sectional design studies have limitations for accurately identifying causes and effects.

## Conclusion

In this study, factors influencing the coping skills of middle-aged adults amid the COVID-19 pandemic included perceived health status, family support, the impact of the event, the underlying disease, and economic status. The explanatory power of the final regression model was 63.0%. Among these influencing factors, the most influential variable was perceived health status. The results of this study can be used as evidence in the clinical field to improve the coping skills of middle-aged adults and to manage and promote health in a situation where COVID-19 infection remains widespread.

The findings of this study are meaningful as the study presents an example that middle-aged adults need a high degree of family support in today's Korean society, which is becoming a nuclear family. Moreover, its notable significance can be used as fundamental data for the development and application of a nursing intervention program that can help improve the coping skills of middle-aged adults amid the COVID-19 crisis.

## Data availability statement

The raw data supporting the conclusions of this article will be made available by the authors, without undue reservation.

## Ethics statement

The studies involving humans were approved by Institutional Review Board of Daejin University (IRB No. 1040656-201912-SB-02-12). The studies were conducted in accordance with the local legislation and institutional requirements. The participants provided their written informed consent to participate in this study.

## Author contributions

Conceptualization and data curation: MG and SS. Methodology, validation, formal analysis, writing—original draft preparation, and writing—review and editing: MG, HW, and SS. Supervision: SS. All authors contributed to the article and approved the submitted version.
